# Why should cognitive neuroscientists study the brain's resting state?

**DOI:** 10.3389/fnhum.2013.00045

**Published:** 2013-02-20

**Authors:** David Papo

**Affiliations:** Center for Biomedical Technology, Universidad Politécnica de MadridMadrid, Spain

Cognitive neuroscience investigates how cognitive function is produced by the brain. Seen from a reverse angle, cognitive neuroscience studies how brain activity is modulated by the execution of cognitive tasks. In the former case, cognitive function is characterized in terms of neural properties associated with the execution of given cognitive tasks, while in the latter it can be thought of as a probe exposing information on brain dynamics.

Brain activity displays dynamics independently of whether a particular task is carried out or not. The question is then: should cognitive neuroscience get interested in the properties of resting brain activity? And, if so, how and to what extent can studying resting brain activity help characterizing the neural correlates of cognitive processes?

## Task-independent cortical activity provides a null condition represented by the brain's properties at rest

In the functional neuroimaging literature, resting state activity is often considered as complementary, in opposition as it were, to task-related brain activity. Insofar as brain activity is essentially defined in terms of time-averaged topography, resting brain activity is associated with patterns of regional blood flow levels. In “cognitive subtraction” neuroimaging studies, where the neural correlates of a particular cognitive function are obtained by identifying differences in regional blood flow activity between tasks differing only in terms of that function, rest is thought of as a *passive null condition*, serving a baseline function against which more controlled cognitive processes can be gauged.

The notion of rest as a passive state has been challenged by functional neuroimaging studies showing that a set of brain regions, comprising medial prefrontal and posterior cingulate cortices, precuneus, inferior parietal and lateral temporal cortices, displays elevated activity at rest and a systematic decreased activity during cognitively demanding tasks (Raichle et al., 2001). The topographical consistency of these decreases led some authors to posit the existence of an *active* organized baseline mode of brain function that would constitute a fundamental brain functioning *default mode* (Gusnard et al., 2001).

While both local activity and functional connectivity at rest have been shown to be altered in various disorders, including schizophrenia, Alzheimer's disease, and autism (Buckner et al., 2008), and to be sensitive to various pharmacological challenges (Anand et al., 2005; Hahn et al., 2007), the functional significance of resting activity for cognitive function is still debated. It has been proposed that the level of resting activity is functionally important, with changes produced by task demands representing just the “tip of an iceberg” (Raichle et al., 2001; Fox and Raichle, 2007). However, it has been argued that not only has rest no impact upon subtractions or other measurements, but there is also no evidence for the level of energy consumption to have implications for cognitive neurosciences (Morcom and Fletcher, 2007).

## Spontaneous activity is not structureless, and is tightly intertwined with stimulus-induced activity

While in the functional neuroimaging literature rest is defined in terms of activation levels and time-averaged topography, electrophysiological studies examined dynamical properties of brain activity at rest and its relationship with task-related brain activity.

Ongoing activity encompasses a set of dynamically switching cortical states, which are continuously reedited across the cortex (Kenet et al., 2003). This re-editing process is not random, but contains rich spatiotemporal structure (Cossart et al., 2003; Beggs and Plenz, 2004; Ikegaya et al., 2004; Dragoi and Tonegawa, 2010). The observed power-law distribution was suggested to indicate that the system self-organizes into a critical state (Chialvo, 2008), where computational capabilities, information transmission and storage, and sensitivity to sensory stimuli are optimized (Haldeman and Beggs, 2005; Kinouchi and Copelli, 2006).

Spontaneous activity massively contributes to the variability observed in stimulus responses (Arieli et al., 1996; Kisley and Gerstein, 1999). Network background activity biases stimulus-induced activity (Azouz and Gray, 1999), and keeps neurons in a permanent state of preparedness for fast responses to variations of synaptic input, allowing neural networks to switch among different regimes of activity (Salinas, 2003). Conversely, environmental demands modulate background oscillations (Pfurtscheller and Lopes da Silva, 1999), and sensory stimuli push ongoing oscillatory activity toward stimulus-specific configurations (Tsodyks et al., 1999).

The relationship between ongoing and task-induced activity goes beyond the level of mutual influence. The repertoire of functional networks utilized by the brain in action is continuously and dynamically active even at rest (Smith et al., 2009). Likewise, population responses to somatosensory stimuli were found to be part of the repertoire of observed spontaneous activity (Luczak et al., 2009), and the spatiotemporal correlations of spikes in the visual cortex to be similar at rest and when observing natural scenes (Fiser et al., 2004). More generally, the dynamic range of a neural network, i.e., the range of stimulus intensities resulting in distinguishable responses (Kinouchi and Copelli, 2006), can be related to its spontaneous activity (Shew et al., 2009). The organization of spontaneous activity reflects past inputs, modulates future network responses (Ohl et al., 2001; Yao et al., 2007), and is predictive of performance on a range of cognitive tasks (Fox et al., 2007; Kounios et al., 2008).

While stimuli can reveal the properties of the system, how the brain explores the various functional configurations representing its dynamic repertoire in the absence of external stimulations is far less understood.

## Resting and task-related activity are two sides of the same coin

The relationship between spontaneous and stimulus-related brain activity can be seen as a manifestation of the fluctuation-dissipation theorem (FDT). The FDT establishes a general relationship between the (non-equilibrium) response of the system to small external perturbations, and the (equilibrium) internal autocorrelation of fluctuations of some observable of the system in the absence of the perturbations (Kubo, 1966). Specifically, the FDT states that, in an equilibrium system, for an observable *X* (e.g., the local amplitude of a signal, or the coupling strength between two brain sites), the ratio between the two-time correlation function *C*_*X*_(*t, t*′) = 〈 *X*(*t*)*X*(*t*′)〉 in the absence of perturbations and the integrated response χ (*t, t*′) = ∫^*t*^_*t*′_ *R*_*X*_(*t*, τ) *d*τ, (where *R*_*X*_(*t* − *t*′) measures how *X* responds at time *t* to a small perturbation at time *t*'), is given by χ (*t, t*′)/*C*_*X*_(*t, t*′) = −1/*T*, where *T* is the system's scalar temperature. Thus, *one may in principle understand the evoked response associated to a stimulus, without applying it, by observing suitably defined correlation properties of brain fluctuations at rest*.

However, resting brain activity does not fulfill the equilibrium requirement of the classical formulation of the FDT, as indicated by its *generic* statistical and dynamical properties typical of non-equilibrium systems, *viz.* non-Gaussian (Freyer et al., 2009), temporal and spatial fractal statistics, i.e., with similar properties at different scales (Novikov et al., 1997; Linkenkaer-Hansen et al., 2001; Gong et al., 2002; Freeman and Barrie, 2000; van de Ville et al., 2010), and intermittent dynamics, with alternating periods of quiet laminar and bursty chaotic activity (Gong et al., 2007), aging and weak ergodicity breaking, a regime where correlations are non-stationary and all possible states are still accessible, but some may require exceedingly long times to visit (Bianco et al., 2007). Conversely, the brain responds to changing external fields not with a single fast relaxation time, but with a series of relaxations spanning a broad range of scales (Lundstrom et al., 2008).

The FDT can be generalized to non-equilibrium systems, taking an explicit form which depends on the shape of the invariant probability distribution toward which the system relaxes (Marini Bettolo Marconi et al., 2008), and even to systems lacking a stationary correlation function (Crisanti and Ritort, 2003; Allegrini et al., 2007).

The way the FDT is violated can be evaluated by plotting χ(*t, t*′) against *C*_*X*_(*t, t*′) (Martin et al., 2001). For equilibrium systems, this yields a straight line with slope −1/*T*. Out-of-equilibrium systems can have a more complex χ − *C*_*X*_ relationship, depending on the particular properties of the system. For instance, multiscaleness and aging lead to a non-linear χ − *C*_*X*_ plot (Crisanti and Ritort, 2003), and a corresponding spectrum of slopes. FDT violations expose task-related brain properties. For example, aging and weak ergodicity breaking predicts maximal brain responses for perturbations with similar properties (Aquino et al., 2011).

## Cognitive function can be described in terms of generic properties of ongoing brain activity

While the notion that cognitive function is a product of neural activity constitutes a firm tenet of modern cognitive neuroscience, the idea that it can be expressed in terms of generic brain properties lies far from the mainstream thinking in the field. Reformulating cognitive function in terms of generic properties of ongoing brain activity has two important implications.

First, *it allows describing complex cognitive processes in which a promoting stimulus has a short-lived or labile influence on observed activity.* For both conceptual and computational reasons, cognitive neuroscience has so far mainly concentrated either on fast event-related relaxational processes, such a perception, or on stationary non-relaxational processes, e.g., memory processes. In the former case, the brain can be considered as a more or less perfectly elastic excitable medium, exiting its stationary quiet state only in the presence of perturbations above a given threshold, which make a transient dynamical cycle observable. Observed activity is typically modeled as a signal blurred by additive uncorrelated Gaussian noise, and the former is extracted from the second by inter-trial averaging, the signal-to-noise ratio improving with the square root of the number of trials. In the latter case, the brain can be treated as a quasi-equilibrium system, and the temporal aspect drastically simplified.

On the other hand, complex phenomena including slow relaxation (e.g., conceptual forms of learning), non-relaxation (e.g., thinking or reasoning), or essentially transient (e.g., mind-wandering) processes, which are in essence subtly modulated ongoing activity, are typically treated as fast relaxational processes, even though fundamental hypotheses on the properties of brain activity (excitability) and of the signal noise (e.g., additivity, Gaussianity, uncorrelatedeness, and absence of memory), do not apply. This supposes methodological procedures (e.g., inter-trial averaging), and experimental adjustments (e.g., smoothing response times, or studying very time-limited event-related parts of a reasoning episode), that limit the variety and dynamical character of cognitive processes that are being studied.

Second, *it affords a wealth of variables through which task-induced reorganization of brain activity may become observable.* Studying task-related brain activity at the long time scales typical of single episodes of learning or reasoning, but also of entire experiments of perceptual phenomena, forces into considering spatiotemporal fluctuations of ongoing activity spanning many orders of magnitude. These fluctuations are not mere noise; rather, they reflect the brain's exploration of its multiscale dynamic repertoire (Ghosh et al., 2007; Deco et al., 2011).

*Ongoing* brain activity may be characterized in terms of known properties such as fractal, non-Gaussian statistics, and intermittency illustrated in the previous paragraph, as well as other properties, e.g., time-reversal symmetry (Gaspard, 2005), path dependence, hysteresis, and hierarchical structure (Sherrington, 2009), which have not yet received the attention of cognitive neuroscientists. This provides a fundamental physical meaning to brain activity, e.g., multifractal fluctuations have a thermodynamic interpretation (Arneodo et al., 1995), while time-reversal asymmetry quantifies the entropy production of the generating mechanism (Gaspard, 2005).

In turn, *task-related* brain activity can be described in as yet non-standard ways as modulations of these properties, e.g., in terms of modulations of probability density functions and the way observed time series converge to these distributions; of symmetries (e.g., scale invariance, time-reversal), so that cognitive processes may differ by a symmetry or a symmetry breaking (Freeman and Vitiello, 2006); of temporal correlations and transitions between different scaling regimes (Buiatti et al., 2007; He et al., 2010; Ciuciu et al., 2012; Zilber et al., 2012), or universality classes, which comprise macroscopic phenomena with the same scaling properties (Bhattacharya, 2009), mirroring dynamical transitions in the system's behavior (Burov and Barkai, 2008); of degree of ergodicity, i.e., ways of visiting the space of possible states; of degree of FDT violation (Cugliandolo et al., 1997) or appropriate correlation functions restoring the FDT (Villamaina et al., 2009); or of information properties and computations (Rabinovich et al., 2006).

*In conclusion*, while the functional interpretation of rest (whether thought as a merely passive condition or, as in the default mode proposal, as an active state) as a baseline remains questionable, theoretical and experimental results suggest that task-related brain activity may be described in terms of generic properties of brain activity at rest. Stimulus-induced exposure of brain functioning and neural characterization of cognition are two sides of the same coin, and the behavioral aspects of cognition are ultimately nothing but macroscopic manifestations of the renormalization of spontaneous brain fluctuations (Fingelkurts et al., 2010). Characterizing task-related brain activity in terms of brain properties at rest allows describing cognitive processes that are either understudied or examined under simplifying assumptions, and quantifying them with a far richer and physically more grounded set of descriptors than has been the case hitherto. The extent to which all possible states of the system can be explored through mere observation of spontaneous brain activity, in experimentally realistic times, or whether stimuli can lead the system to otherwise unexplored states are still unexplored issues, which will require further investigation of the properties of systemic brain activity.
